# Mandibular Flexure and Crestal Bone Stress Distribution on an Implant-Supported Fixed Full Arch Mandibular Prosthesis: Finite Element Analysis in Three Dimensions

**DOI:** 10.7759/cureus.39357

**Published:** 2023-05-22

**Authors:** Suraj Sharma, Shashikala Jain, Himanshu Gupta, Sai Govind Gavara, Pratibha Panwar, Ramanjeet Kaur Grover

**Affiliations:** 1 Department of Prosthodontics, Crown and Bridge and Oral Implantology, Maharaja Ganga Singh Dental College and Research Centre, Sri Ganganagar, IND

**Keywords:** mandibular flexure, total deformation, von mises stress, tilted implants, 3d fea

## Abstract

Aim

This study's objective was to assess and analyze, using 3D Finite Element Analysis, the impact of four mandibular complete arch superstructures on the distribution of stress in the crestal bone during mandibular flexure.

Materials and methods

Four Finite element models of the mandible with different implant-retained framework designs have been developed. Three of these models had six axial implants placed at intervals of 11.8 mm, 18.8 mm and 25.8 mm from the midline, respectively. One model had two tilted implants and four axial implants splinted with a single piece of framework at intervals of 8.4 mm, 13.4 mm and 18.4 mm from the midline. For analyzing the stress distribution, the finished product was transferred to ANSYS R 18.1 software (Sirsa, Haryana, India) for finite element simulation, the models were constructed, the ends were restrained, and bilateral vertical loads of 50N, 100N and 150N were applied to the distal part of the framework.

Results

Bilateral loads were applied to each of the four 3D FEM and after assessment of Von Mises Stress and Total Deformation, a finding was made that the model with six axial implants supported by a single piece of framework underwent the highest total deformation and the model with four axial implants and two implants with distal tilts displayed most significant Von Mises stress.

Conclusion

Within the constraints of this 3D FEA, it was determined that mandibular flexure and peri-implant bone stress were affected by the way the framework is divided and the nature of mandibular movement. The three types of frames with the least bone stress are demonstrated by the mandibular deformation that results from two-piece frameworks on axial implants. Regardless of the number of implants, the single framework splinted with six implants shows a flexure in mandible with the highest bone stress around the implant irrespective of the angulation of the implant.

Clinical significance

When it comes to edentulous jaws, reducing stress in implant-supported restorative systems at varying degrees of the bone and implant interfaces and superstructures of prosthetics is one of the fundamental goals of implant treatment. A framework with proper design and a low modulus of elasticity reduces mechanical risk. Additionally, a larger number of implants helps to prevent cantilevers and spacing between the implants.

## Introduction

The science of implantology has advanced greatly since Dr. Branemark first introduced it to the world of dentistry. Since the middle of the 19th century, researchers have looked into how mechanical stress affects bone tissue. The anatomical structure of the human mandible results in complex elastic biomechanical behavior under functional load. When a person has natural teeth and no prosthetics the stress-strain results agree with Frost's mechanostat theory (1500-3000 microstrains) [1]. However, when a person's entire mandible is missing and has been replaced with a fixed prosthesis or an implant-supported prosthesis, a rigid structure is made which has a lever effect that alters how bone stress and strain are distributed as the mandible flexes [2]. In order to create 3D models, the system domain is divided into a number of considerably more manageable and straightforward domains using FEA.

Early loss of posterior teeth may lead to alveolar bone loss, pneumatization of the maxillary sinus in the maxilla, and surfacing of the mandibular nerve, hence in the posterior region implants should not be placed immediately. An alternative would be to employ tilted implants, in areas where the height of the bone would not permit the placement of implants. According to studies by Zampelis et al. (2007) [3] and Bevilacqua et al. (2008) [4], it maximizes the utilization of the present bone and permits implantation of a fixed prosthesis that has least amount of cantilever.

An important factor in determining whether a dental implant will succeed or fail is the process of stress transfer to the surrounding bone. The loading protocol, interface of bone and implant, the length and width of the implants, the dimensions and qualities of the implant surface, the kind of prosthesis, and the volume and standard of the surrounding bone all affect how much load is shifted from the implants to the nearby bone. The implant's contact area with the mandibular and maxillary bone can experience stress distribution that can be predicted using FEA [5].

The stress distribution and mandibular deformation brought on by a variety of loads applied bilaterally in various prosthesis, whether the design is supported with implants and supported distally by axial and tilted implants or cantilevered were analyzed using finite element models of various superstructure designs in the current study.

## Materials and methods

Four 3D FEM models of the human mandible were made on a computer platform for the current in vitro study using the program CATIA version V5 R20 (Sirsa, Haryana, India).

Model designing

**Figure 1:**
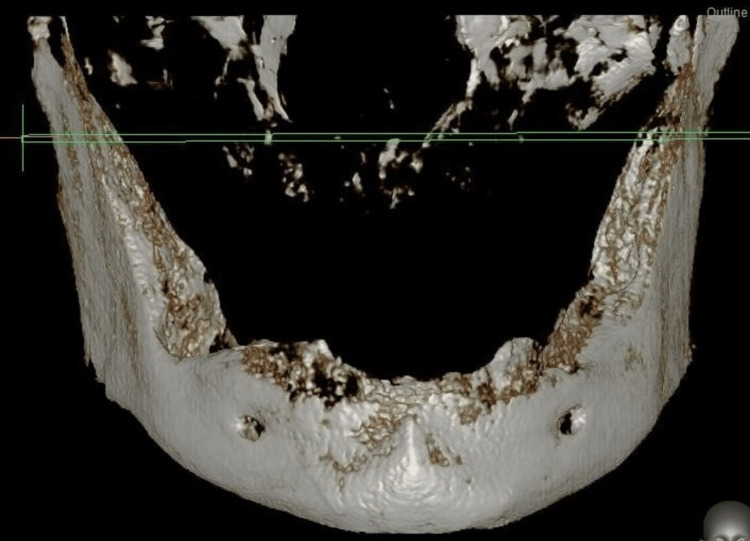
3D scanned image of the human mandible with Cone-beam CT-Vatech Smart Plus

By using Cone-beam CT-Vatech Smart Plus (Vatech India Pvt. Ltd, New Delhi, India) to 3D scan the human jaw, the geometry of the edentulous mandible was identified. An edentulous human skull anatomical model was used to build a computed tomography of a mandible, which was then replicated to make a three-dimensional model. Images were captured using brief slice intervals of 0.5 mm increments. A Finite element structure of the mandible which was three-dimensionally oriented was built using the software CATIA version V5 R20 (Sirsa, Haryana, India) after slices were placed together (Figure 1).

**Figure 2:**
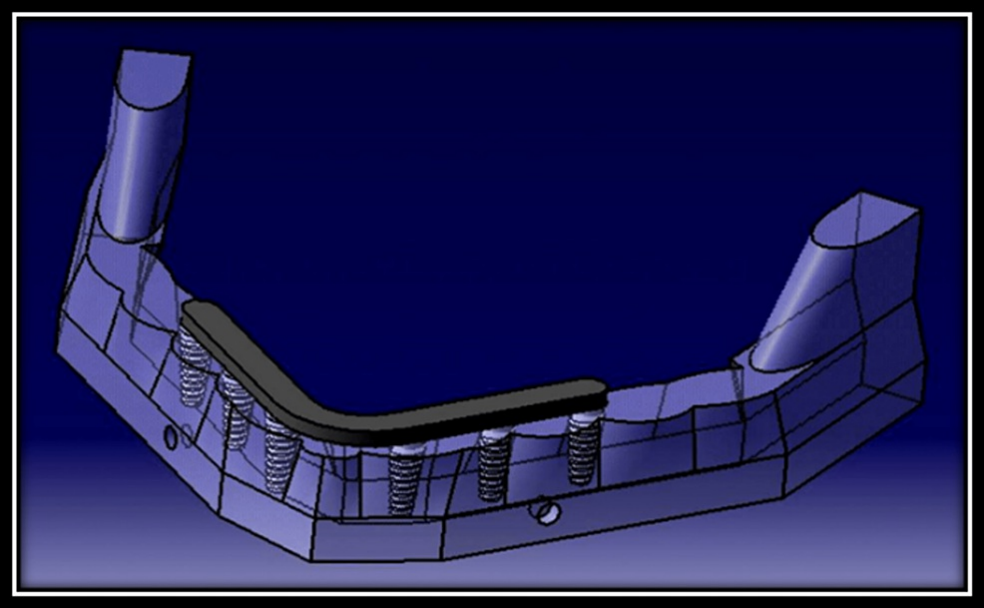
Finite element model of one-piece framework-splinted mandibular six implants

The mandibular bone height in the area of the symphysis was 24 mm, and the intercondylar dimension was 119 mm. CATIA V5 R20 was used to design the finite element (FE) and solid (3D) models for the implants. The model included solid cylindrical titanium implants exhibiting a total diameter of 4 mm and a length of 11 mm. In total, four implant-retained mandibular restorations with various frameworks were created:

Model 1: One-piece framework by splinting six axial implants (Three implants were placed 11.8 mm, 18.8 mm and 25.8 mm each side from respective midline) (Figure 2).

**Figure 3:**
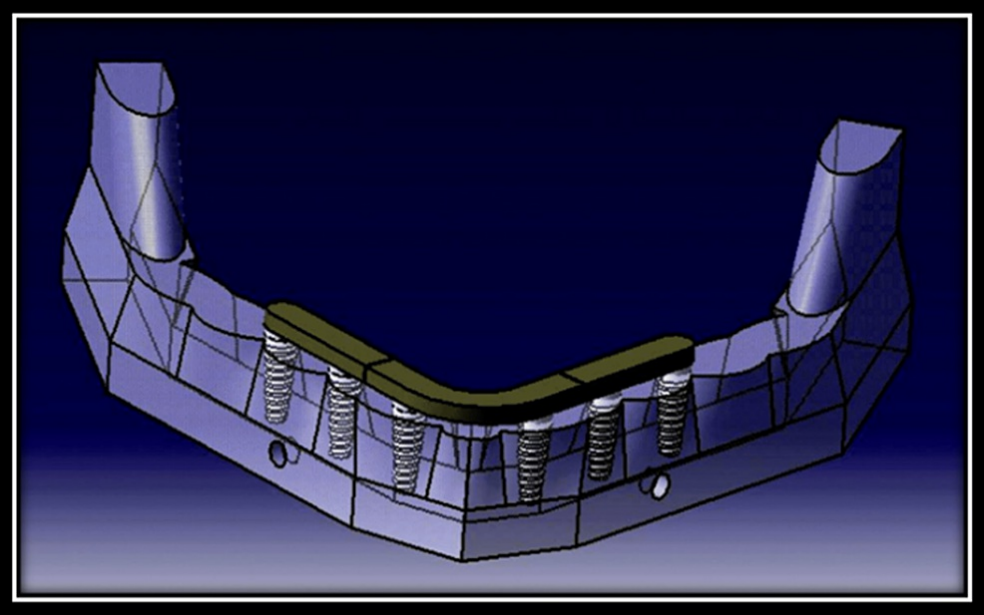
Two posterior and one anterior portion in a three-piece framework on six axial implants

Model 2:** **Two posterior and one anterior portion in a three-piece framework on six axial implants (The implants were placed as in model 1) (Figure 3)*.*

**Figure 4:**
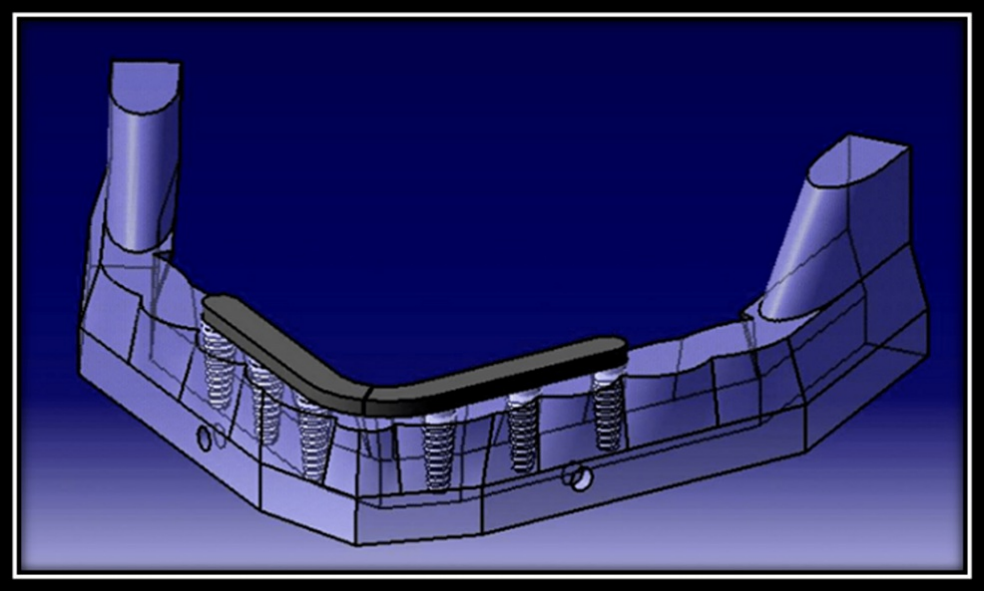
Two-piece framework divided along the midline on six axial implants.

Model 3:** **Two-piece framework divided along the midline on six axial implants (The Implants were placed as in model 1) (Figure 4).

**Figure 5:**
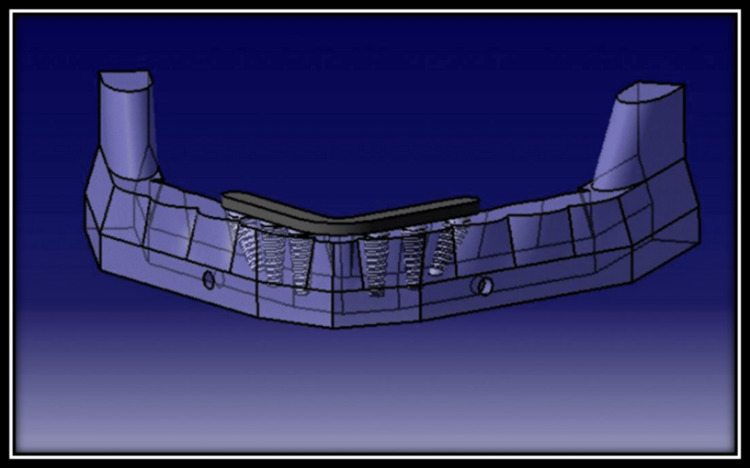
One-piece framework by splinting two tilted implants and four axial implants.

Model 4:** **One-piece framework by splinting two tilted implants and four axial implants (On each side, three implants were positioned 8.4 mm, 13.4 mm, and 18.4 mm from the midline, respectively) (Figure 5). The distal-most implants were 30° inclined to the occlusal plane.

For mesh refinement in the Finite element model, the cortical and trabecular bones are assumed as having complete osseointegration with the implant surface. Then, the finished product was transferred to ANSYS R 18.1 (Sirsa, Haryana, India) for finite element simulation. The average occlusal force was calculated from the literature to be 50N, 100N and 150N. Bilateral load on the distal half of the framework was applied in order to quantify the distribution of stress. Constraints at the ends of bone segments and the application of force on top of the Co-Cr framework are utilized to approximate the complex balance between masticatory forces and their reactions.

Mesh generation

**Figure 6:**
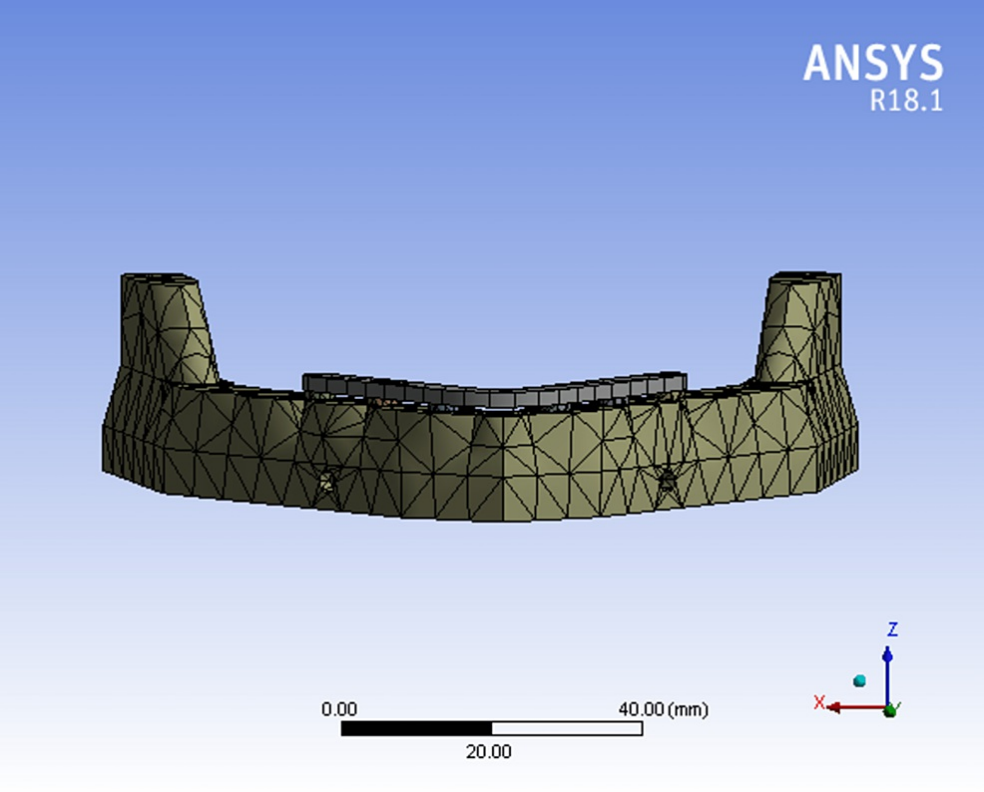
Mesh model of bone mandible and six implants supported with a one-piece framework.

**Figure 7:**
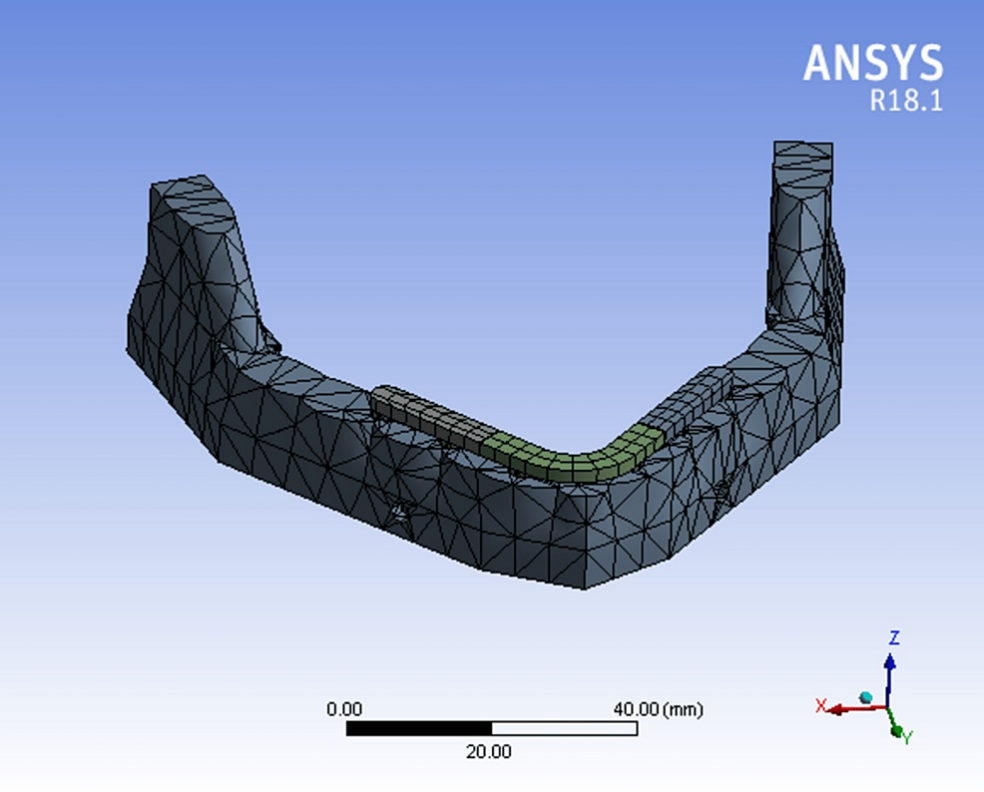
Mesh model of bone mandible and six implants supported with a three-piece framework.

**Figure 8:**
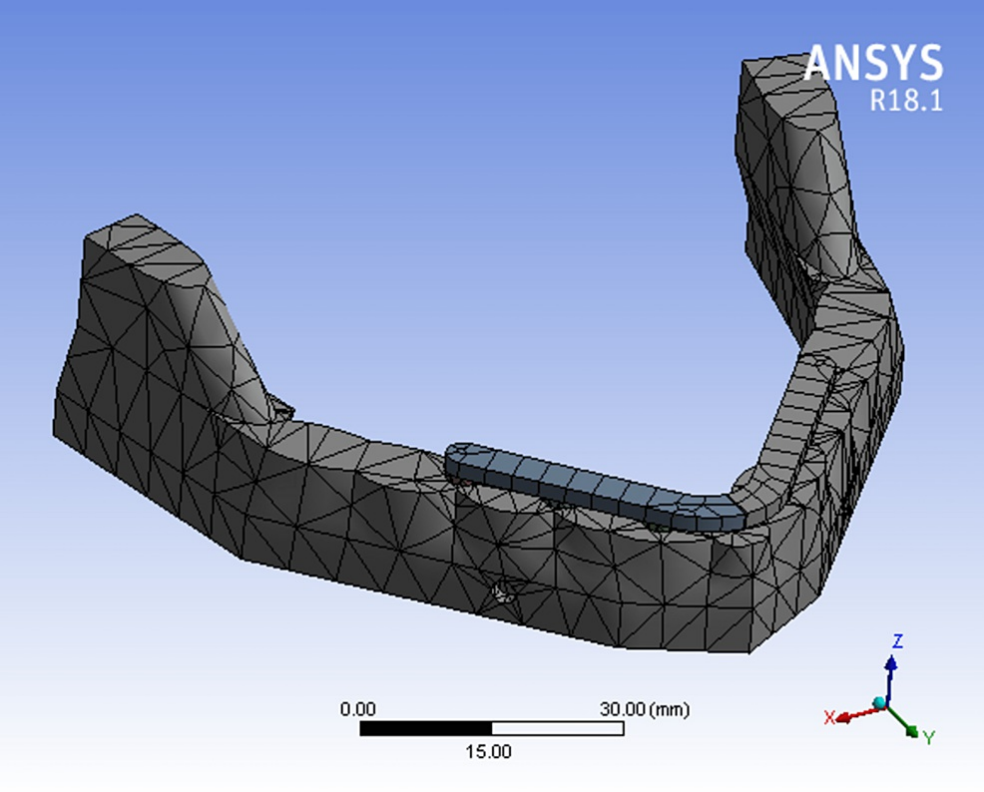
Mesh model of bone mandible and six implants supported with a two-piece framework.

The ANSYS version 19.0 software (Sirsa, Haryana, India) was used to transfer the designed models. Each model's geometry was broken down into a number of distinct subregions, or "elements" which were joined at distinct nodes (Figures 6-8). Some of these components have predetermined displacements, while others have predetermined loads (Table 1).

**Table 1:** Number of elements and nodes of the 3D Finite element models.

Models	Nodes	Elements
Model 1 (FIG. 6)	34018	18547
Model 2 (FIG. 7)	34320	18580
Model 3 (FIG. 8)	34107	18555
Model 4 (FIG. 9)	37792	21026

Assigning the material properties

All substances in relevance were considered isotropic, homogeneous, and linearly elastic. The cortical and cancellous bones were modelled to have bone of the D2-type elastic characteristics. The relevant elastic properties, such as Young's modulus (E) and Poisson's ratio (µ), were calculated utilizing the literature (Table 2) [6].

**Table 2:** Material properties for the FEA model. FEA: Finite element analysis

Material	Young’s Modulus (E) (GPa)	Poisson's Ratio (µ)
Titanium (Abutment, implant)	110	0.35
Cancellous/Spongy bone	1.37	0.3
Cortical bone	13.7	0.3
Co-Cr alloy (framework)	218	0.33

Boundary conditions

**Figure 9:**
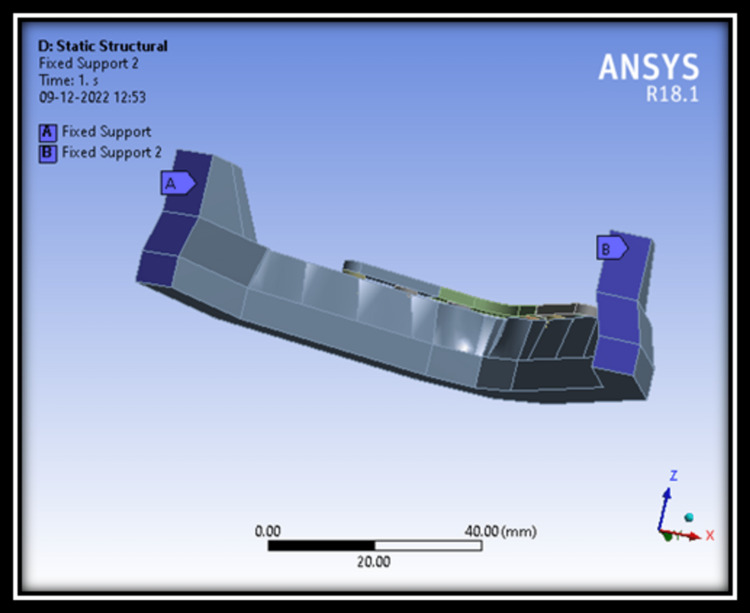
Finite element model showing fixed loads/end constraints applied on the posterior end of mandible.

It has been found common during the construction of finite element model of the jaw bone to apply fixed constraints. Using constraints at the ends of bone segments and load on top of the superstructure, the intricate balance between forces in the mouth and their effects is roughly approximated (Figure 9).

Loading condition

A functional load of 50N, 100N and 150N was applied to each model, respectively.

Types of solution

The FEA model assumed that osseointegration was in its ideal state, implants were assumed to be 100% osseointegrated and node-to-node connection was considered at the bone-implant interface. To examine how different cross arch superstructures affect mandibular flexure, the Finite Element analysis of each design was performed using ANSYS (version R 18.1).

Von Mises Stress, Strain and Degree of deformation were recorded and utilized to sum up the effect of occlusal loads on crestal bone stress distribution and mandibular flexure on an implant-supported fixed full arch mandibular prosthesis.

## Results

The current study examined and assessed the mandibular flexure and a fixed mandibular prosthesis supported by implants that distribute the crestal bone stress. Four 3D models were designed and 50N, 100N and 150N forces were applied on each model and Von Mises Stress, strain and degree of deformation were recorded.

**Figure 10:**
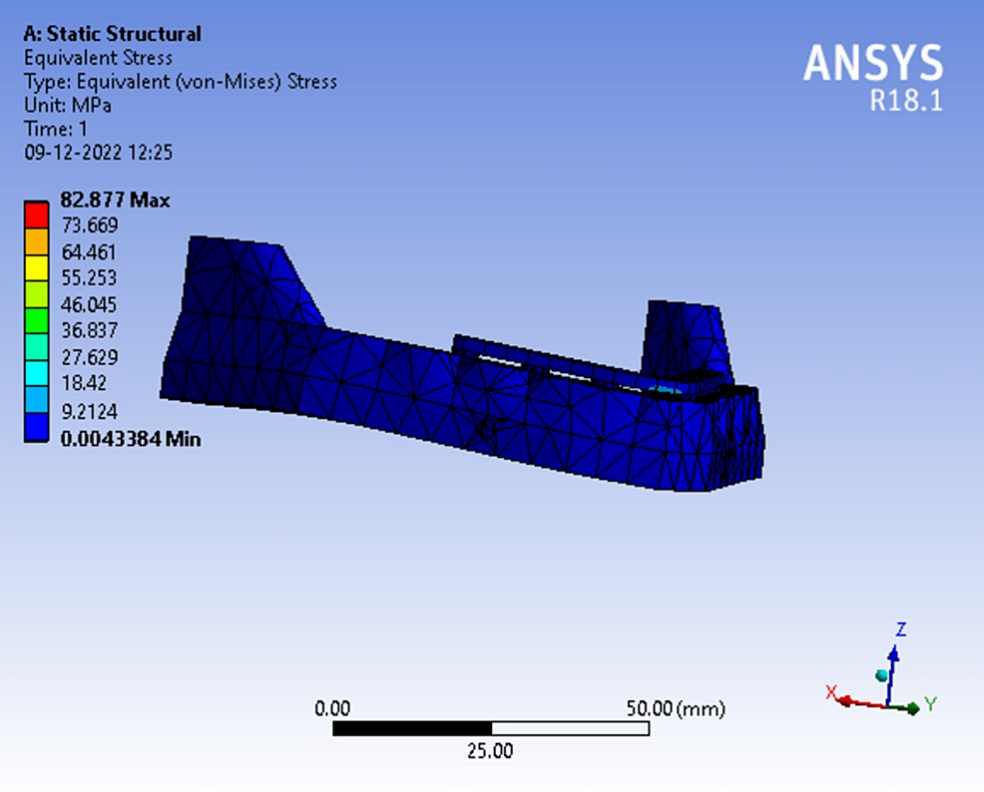
Assortment of stress in peri-implant bone in single piece framework.

On application of 50N load, Figure 10depicts Equivalent Von Mises stress concentrated at the labial surface of the superstructure above the mandibular symphysis region in light blue color which was recorded from the scale adjacent to it that gradually increased to 82.877 MPa*.*

InFigure 11*, *where the superstructure was divided into three halves Von Mises stress was concentrated more in the lingual surface of the framework at the mandibular symphysis region which was measured to be 82.703 MPa.

**Figure 11:**
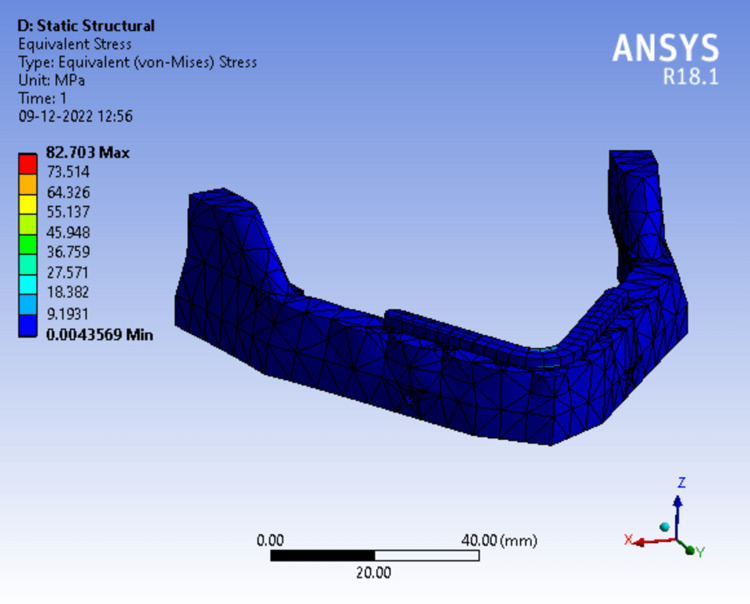
Assortment of stress in peri-implant bone in a 3-piece framework.

**Figure 12:**
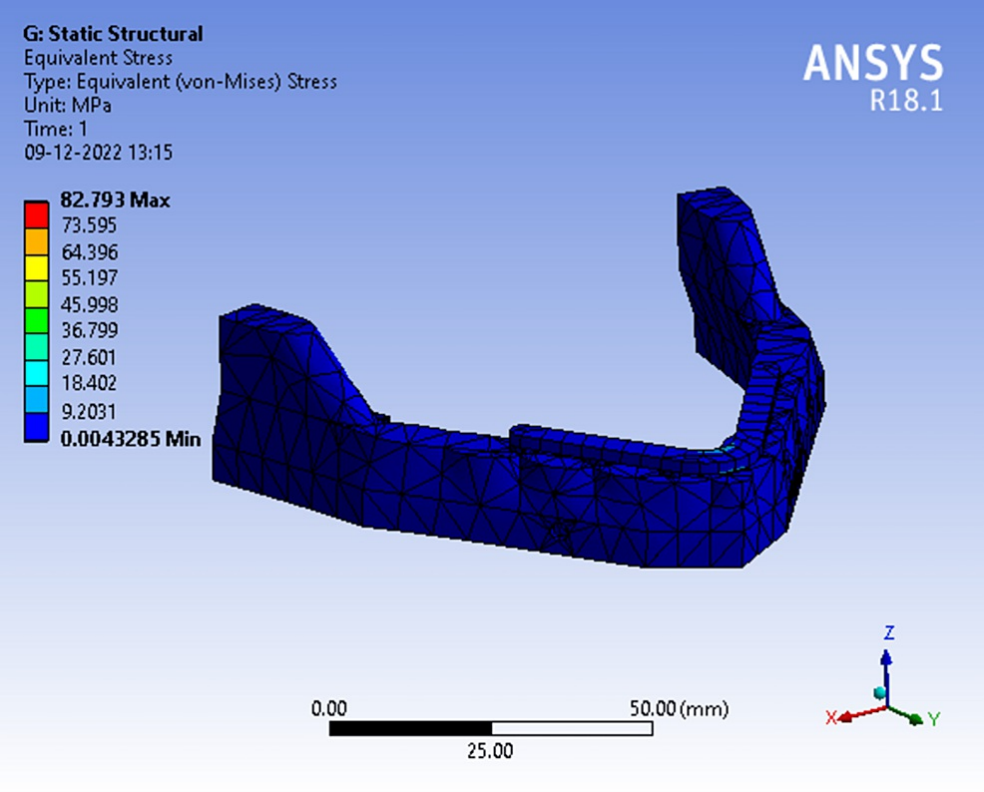
Assortment of stress in peri-implant bone in a 2-piece framework.

In Figure 12, the framework was divided from the midline where the stress was concentrated around the lingual region of the framework above the symphysis which was recorded 82.793 MPa.

**Figure 13:**
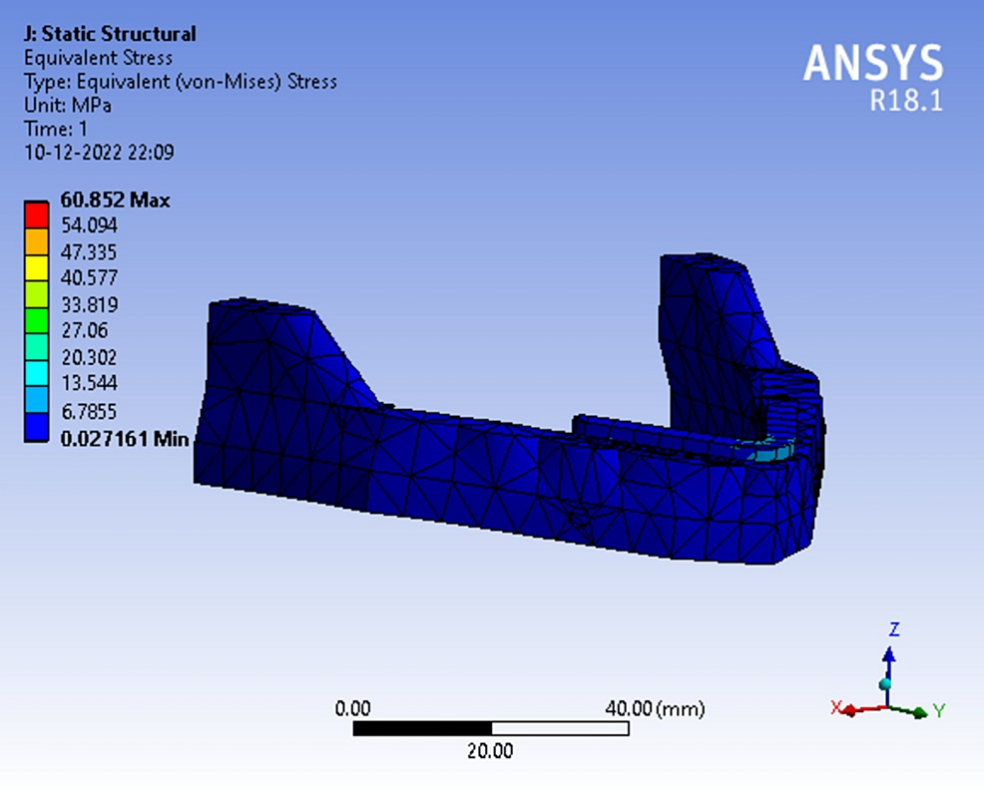
Assortment of stress in peri-implant bone in a single-piece framework splinted with tilted implants.

In Figure 13 (Model 4), the Von Mises stress (60.852 MPa) was least generated and concentrated around the lingual and labial region of the framework.

**Figure 14:**
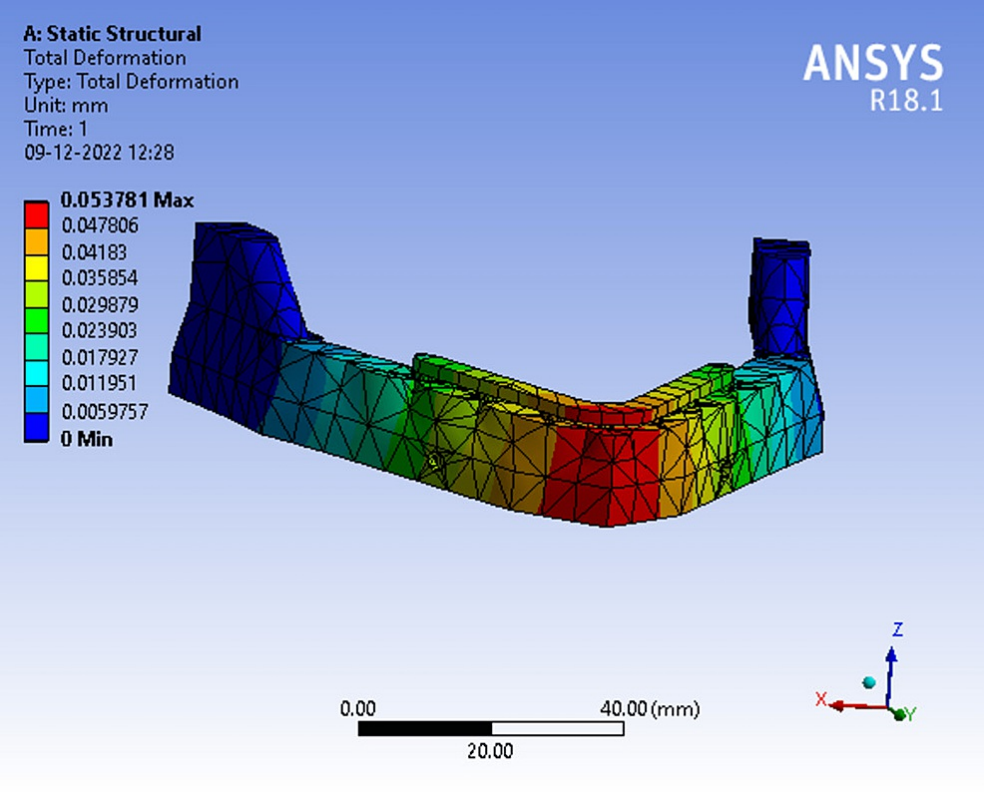
Assortment of total deformation in peri-implant bone in a single-piece framework.

While assessing the total deformation in Model 1 (Figure 14) at 50N load the red zone indicates areas of highest deformity which was seen at the mandibular symphysis region gradually dissipating towards the ramus. The readings were measured from the scale adjacent to the analysis and maximum deformation was recorded.

**Figure 15:**
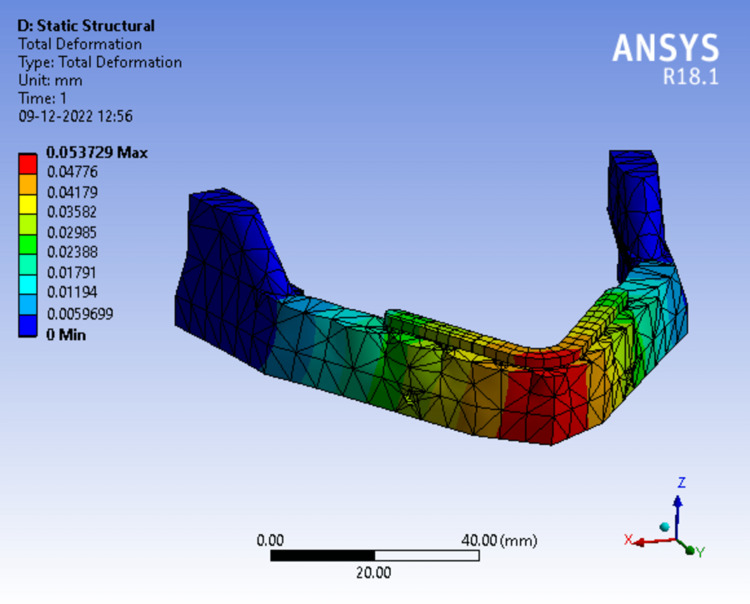
Assortment of total deformation in peri-implant bone in a 3-piece framework.

Assessing the total deformation in model 2 (Figure 15) at 50N load, the red zone indicates areas of highest deformity which was seen at the mandibular symphysis region which gradually dissipates towards the ramus. The readings were measured from the scale adjacent to the analysis and maximum deformation was recorded.

**Figure 16:**
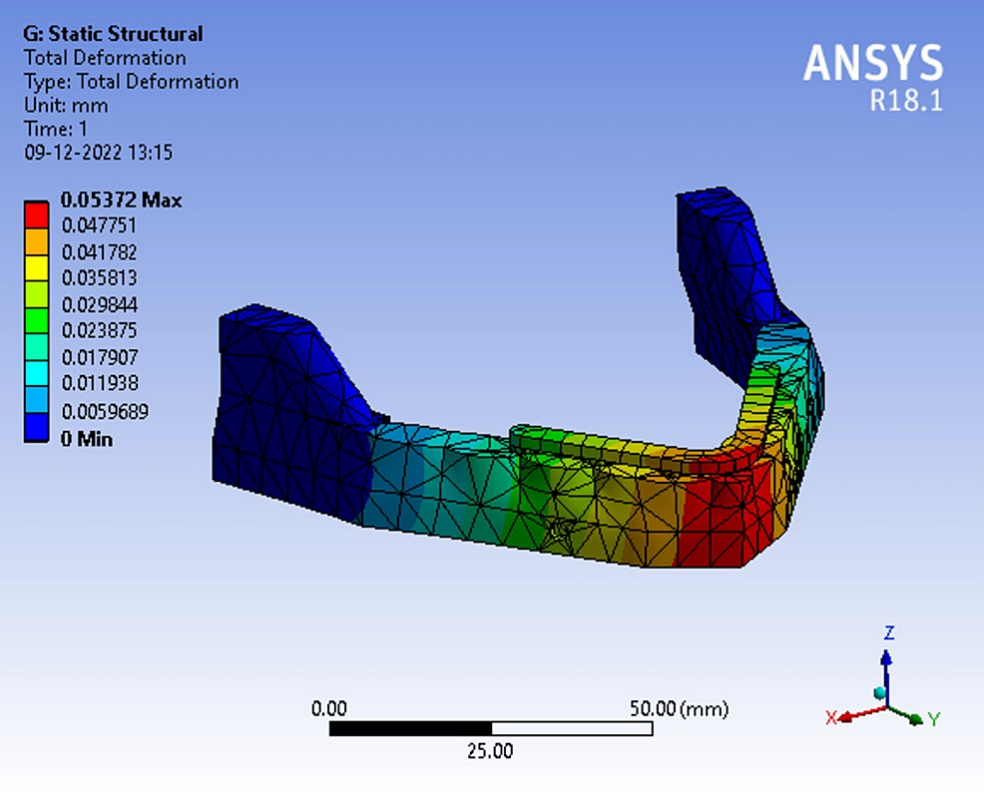
Assortment of total deformation in peri-implant bone in a 2-piece framework.

Figure 16, depicts total deformation in model 3 at 50N load the red zone indicates areas of highest deformity which was seen at the mandibular symphysis region which gradually dissipates towards the ramus. The readings were measured from the scale adjacent to the analysis and maximum deformation was recorded.

**Figure 17:**
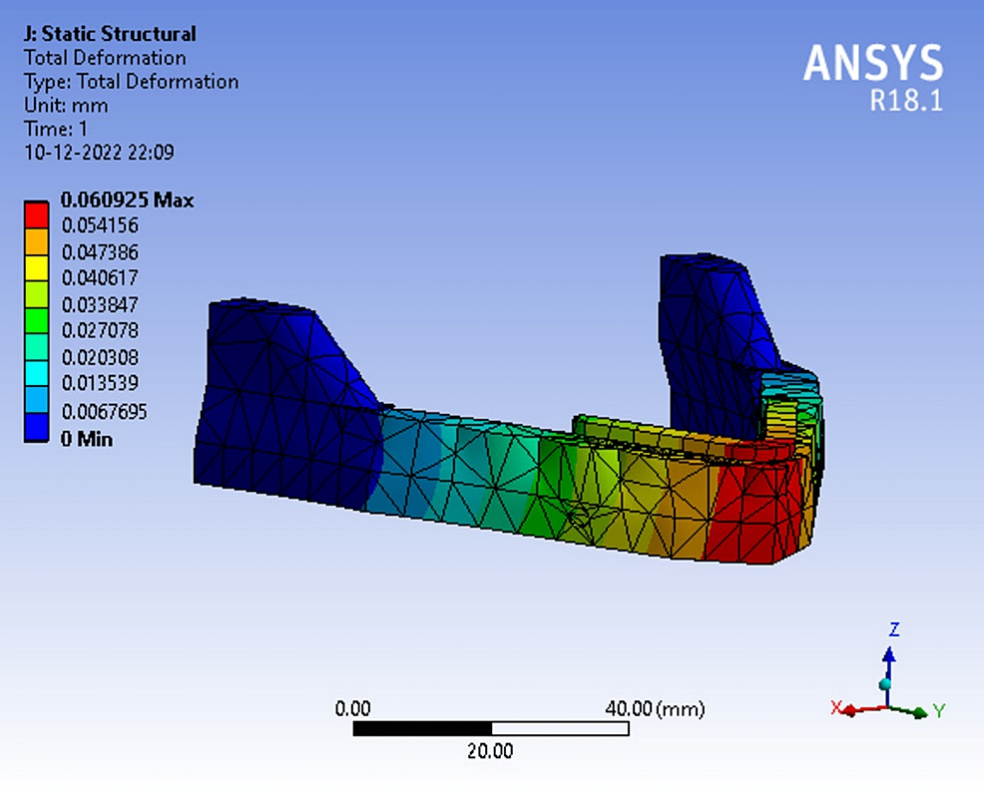
Assortment of total deformation in peri-implant bone in a single-piece framework splinted with tilted implants.

In Figure 17, total deformation in Model 4 was observed. The red zone indicates the area of highest deformity at the mandibular symphysis region. The readings for maximum deformation were recorded.

Tables 3-5 show the evaluation and comparison of Von Mises stress, equivalent strain, and total deformation on applying bilateral load of 50N, 100N and 150N respectively on different mandibular models.

**Table 3:** Evaluation and comparison of Von Mises Stress, Equivalent Strain, and Total Deformation on applying a bilateral load of 50N on different mandibular models.

Model	Von Mises Stress (MPa)	Equivalent strain	Total deformation (mm)
Model 1	82.877	0.00076850	0.053781
Model 2	82.703	0.00076689	0.053729
Model 3	82.793	0.00076772	0.053720
Model 4	60.852	0.00069898	0.060925

**Table 4:** Evaluation and comparison of Von Mises Stress, Equivalent Strain, and Total Deformation on applying a bilateral load of 100N on different mandibular models.

Model	Von Mises Stress (MPa)	Equivalent strain	Total deformation (mm)
Model 1	165.75	0.0015370	0.10756
Model 2	165.41	0.0015338	0.10746
Model 3	165.59	0.0015354	0.10744
Model 4	121.70	0.0013980	0.12185

**Table 5:** Evaluation and comparison of Von Mises Stress, Equivalent Strain, and Total Deformation on applying a bilateral load of 150N on different mandibular models.

Model	Von Mises Stress (MPa)	Equivalent strain	Total deformation (mm)
Model 1	248.63	0.00230550	0.16134
Model 2	248.11	0.00230070	0.16119
Model 3	248.38	0.0023032	0.16116
Model 4	182.56	0.0020970	0.18278

**Figure 18:**
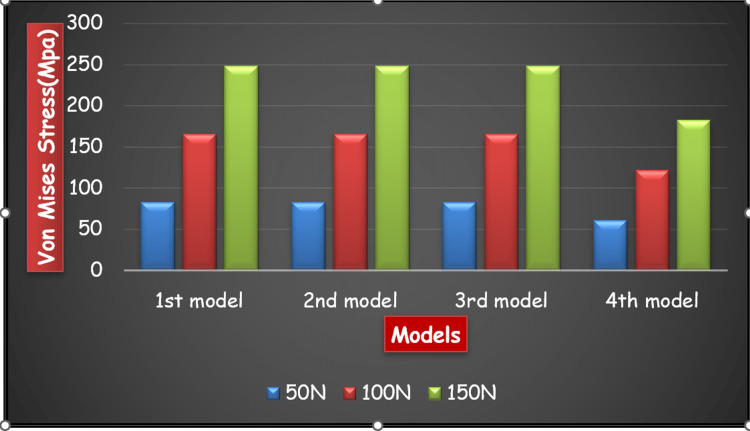
Comparison of Maximum Von Mises stresses on the application of a load of 50N, 100N, and 150N on Models 1, 2, 3, and 4 accordingly.

**Figure 19:**
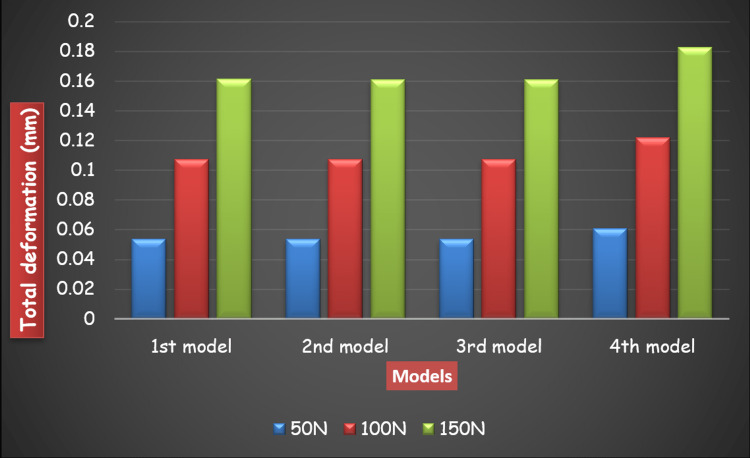
Comparison of total deformation on the application of a load of 50N, 100N, and 150N on Models 1, 2, 3, and 4 accordingly.

Figure 18 and Figure 19 show a comparison of Von Mises stress and total deformation on each model under different loading forces.

## Discussion

An algorithmic technique for analyzing structures is the finite element method. With the aid of algebraic equations that a computer has solved, a three-dimensional model can be recreated using finite element analysis (FEA). Static and dynamic analyses are two different forms of FEA [7]. A dynamic study might be necessary if larger mandibular velocities are present, as it might happen when someone unintentionally bites down on something hard. The simulation of clenching, grinding, and masticatory circumstances is thought to be appropriate for static analysis. For the purpose of this study, static loads are thought to be enough. The ability of various attachment systems to distribute stress using different bar designs is the subject of a very small number of investigations. The current investigation concentrated on the pressures and changes around the peri-implant bone and mandibular flexure by using six standard implants splinted with various superstructure designs.

For this study's purpose, four 3D geometric models that simulate an edentulous human mandible were constructed using the 3D CATIA program (version V5, R20) (Sirsa, Haryana, India). These models were later created as 3D FEM models by utilizing the above-mentioned CATIA software. ANSYS software was used to analyze the stresses on each model. Four Finite element models were created from which three models had six axial implants splinted with a single piece of framework, three pieces of a framework with one anterior and two posterior portions, two pieces of framework divided along the midline, and the fourth model had two distally placed implants that were inclined at an angle of 30° to the crest of the ridge (Malo et al., 2010) [8].

The properties used for the designing of the framework were of Co-Cr alloy because according to Padhye et al. (2015) [6], the cancellous bone, cortical bone and titanium implants all bore the least amount of stress with the Co-Cr framework bearing the most. Clinical long-term studies on the use of polyetheretherketone (PEEK) as a fixed implant-supported full arch framework are rare. However, full-contoured Monolith Zirconia has a higher modulus of elasticity and low density in comparison to Co-Cr alloys but due to its high cost and less usage by dental laboratories, the Co-Cr framework is preferred in this study. The mechanical properties of Co-Cr include high rigidity (high elastic modulus), high strength, excellent bonding with ceramic, corrosion resistance, and low cost.

According to published research, in the molar region, the masticatory forces vary from 50N to 150N [9]. As a result, following modeling the first molar tooth region was subjected to bilateral static loads of 50N, 100N and 150N.

In terms of overall stress distribution, according to Vafaei et al. (2011) [10], Satpathy et al. (2015) [11], and da Silva et al. (2010) [12], the bar attachment method appears to function superior compared to ball/O-ring attachment technique as the pressures are transferred more uniformly. As a result bar attachments were chosen for the investigation. Implant splinting was justified on the grounds that greater prosthesis stability would reduce strains.

For comparison of peri-implant bone stresses and mandibular flexure in different models, Von Mises equivalent stresses were used because they are typically used in FEA studies to provide a comprehensive overview of the site's stress status [13,14]. The information in Tables 3-5 indicates that the location of the implants and the type of prosthetic superstructure are at least two of these criteria that have a considerable impact on the flexibility of the implant-restored mandible. In Model 3, when the framework is divided from the midline without moving the implants a cantilever is created. Cantilever has a negative impact on the biomechanics of implant restorations, according to Goodacre et al. (2003) [15]. The quantity of strains on all structures (implant, prosthetic and bone: cortical and cancellous) rose uniformly at every 5 mm increment of the cantilever length.

When all the three loads, i.e. 50N, 100N and 150N, were applied on all the four frameworks, Model 3, i.e., two-piece framework with a midline split on six axial implants showed the least total deformation with minimal mandibular flexure and peri-implant bone stress, followed by Model 2, i.e., two posterior and one anterior portion in a three-piece framework on six axial implants followed by Model 1, i.e., one-piece framework by splinting six axial implants even when the maximum load was applied. Therefore, Model 3, i.e. two-piece framework with a midline split on six axial implants, is the best among all the four models. Model 4, i.e. one-piece framework by splinting two tilted implants and four axial implants, showed maximum deformation among all the four models even when the minimum load was applied.

Von Mises stress: Model 1 > Model 3 > Model 2 > Model 4

Equivalent Strain: Model 1 > Model 3 > Model 2 > Model 4

Total Deformation: Model 4 > Model 1 > Model 2 > Model 3

Clinical implications

All four models have a potential use in the clinical practice in atrophic mandibular jaws. Increasing the number or the diameter of the implants may affect implant survival in a positive manner. In the posteriorly atrophic mandible, the use of tilted implants may be preferred for long-term success, especially when the narrow diameter implants need to be inserted.

As the present study is a static FEA study on mandibular flexure and peri-implant bone stress it is not possible for a mathematical/computational model to reproduce as exactly as possible all the biological characteristics to simulate muscular activity patterns. Considering the aforementioned simplification and assumptions, the distribution data of this study has to be understood in qualitative terms rather than in quantitative terms.

This study somewhat agrees with the dynamic analysis conducted by Martin-Fernandez et al. (2018) [2]. This study's scope is significant in accomplishing structural optimization in support of the most successful experimental model.

## Conclusions

Within the limitation of the study, it can be concluded that peri-implant bone stress and mandibular flexure are influenced by the division of the framework and the kind of mandibular movement. Mandibular deformation caused by a two-piece framework on axial implants exhibits the least amount of bone stress. Regardless of the implant's angulation, the complete framework exhibits the highest peri-implant bone stress.
